# Early life diarrhoea and later blood pressure in a developing country: the 1982 Pelotas (Brazil) birth cohort study

**DOI:** 10.1136/jech.2008.077818

**Published:** 2009-01-06

**Authors:** G D Batty, B L Horta, G Davey Smith, F C Barros, C Victora

**Affiliations:** 1 MRC Social & Public Health Sciences Unit, University of Glasgow, Glasgow, UK; 2 Post-Graduate Programme in Epidemiology, Universidade Federal de Pelotas, Pelotas, Brazil; 3 Department of Social Medicine, University of Bristol, Bristol, UK; 4 PAHO/WHO Latin American Centre for Perinatology, Montevideo, Uruguay

## Abstract

**Background:**

It has recently been hypothesised that acute dehydration in early childhood may “programme” increased blood pressure via salt retention. We examined whether there was an association between episodes of diarrhoea (a proxy for acute dehydration) and later measured blood pressure.

**Methods:**

In the 1982 Pelotas birth cohort study (Brazil), parents/carers reported hospital admissions for diarrhoea in the first 12 and 20 months of study members’ lives. Blood pressure was subsequently measured directly in adolescence (aged 15, 18, 19 years) and early adulthood (aged 23 years).

**Results:**

We found no evidence of an association between diarrhoea in the first 12 months of life and blood pressure measured at any point in adolescence or early adulthood. These findings were unchanged after adjustment for a range of covariates. Equally null results were apparent when diarrhoea admissions in the first 20 months of life, access to home sanitation and use of piped water were the exposures of interest.

**Conclusions:**

Early life proxies for dehydration and diarrhoea were unrelated to later blood pressure in this examination, the most comprehensive to date, of the potential association.

It has recently been suggested that dehydration in infancy, most likely resulting from an episode of diarrhoea, will subsequently lead to a permanent elevation in blood pressure in humans.^1^ This hypothesis is based on the proposition that dehydration could “programme” increased water, and hence salt, retention in a biological attempt to protect against the threat of future life-challenging dehydration.^1^ While this may have positive implications for survival in the short term, given that salt retention gives rise to elevated blood pressure,^2^ this would be disadvantageous in the longer term owing to an increased risk of hypertension and cardiovascular disease, most obviously in low- and middle-income countries where diarrhoea is prevalent.

The few empirical tests of this hypothesis have been inconclusive. UK children who had a hospital record of dehydration in the first 6 months of life had raised diastolic but not systolic blood pressure.^1^ Further, in a study that utilised climate data as an instrumental variable for infant dehydration, infants who were exposed to conditions that were more likely to precipitate episodes of diarrhoea (summers with higher temperature and lower rainfall) had increased levels of systolic blood pressure in older age.^3^ However, three similarly designed UK birth cohort studies^4^^–^6 revealed concordant findings: the blood pressure and/or coronary heart disease risk of middle- to older-aged people did not differ between those with and those without a nurse-recorded early life episode of diarrhoea.

Several of these studies are characterised by a low number of cases of dehydration or diarrhoea, which hampers data interpretation. The Pelotas study,^7^ which, usually, has repeat measurement of blood pressure across the life course, is based in southern Brazil in an environment where episodes of diarrhoea among the young are somewhat more common than in northern Europe, and therefore does not have this limitation.

## METHODS

The 1982 Pelotas birth cohort study has been described in detail elsewhere.^7^ In brief, in 1982, the city’s five maternity hospitals were visited daily by project workers and all 5914 live births were included in the study. Subsequently, there have been eight follow-up studies of cohort members. Of those pertinent to the present analyses, in 1983 the mothers/carers of a subgroup of infants (N = 1457; 79.3% of surviving target population) who were 12 months old at the time (ie, born January to April 1982) were asked about episodes of illness in the preceding period, including diarrhoea, that required hospital admission. Questions regarding domestic sanitation and piped water were also posed. In 1984, around 20 months after birth, attempts were made to re-contact all study participants and their parents/carers (N = 4934; 87.2% follow-up) and the same enquiries were made. In a validation study involving 120 cohort members, hospital records and parent/carer reports were grouped into broad underlying causes: diarrhoea, pneumonia, asthma/bronchitis, surgery and other.^8^ Agreement was high between the two sources of information, varying between 85% (pneumonia) and 97% (diarrhoea).^8^

Blood pressure measurement, together with height and weight, was taken in 1997 when the cohort members were aged approximately 15 years (subgroup of N = 1076; 71.8% of a 27% systematic target sample); in 2000 when aged approximately 18 years (subgroup of N = 2250; 78.9% of an all-male target sample); in 2001 when aged approximately 19 years (subgroup of N = 1031; 69% of a 27% systematic target sample); and, finally, 2004/5 when aged approximately 23 years (N = 4297; 77.4% of all apparently surviving study members). In the earlier home visits, blood pressure was measured twice, at the beginning and at the end of the interview, in the sitting position with a calibrated aneroid sphygmomanometer with appropriate cuff size. In the 2004/5 visit, blood pressure was again recorded twice but using an Omron digital portable wrist monitor (Omron, Beijing, China). The mean of the two readings were used in the present analyses.

## RESULTS

Using multiple linear regression we compared the blood pressure of study members who had experienced diarrhoea in the first 12 months of life with those who had not (table 1). The period prevalence of diarrhoea admissions varied between 2.8% and 6.5% depending on the analytical sample under consideration. There was no evidence that infant diarrhoea was linked to an increase in blood pressure for any of the four measurements. The suggestion of a reverse effect—an elevated blood pressure level at age 19 years in children who had not experienced an admission due to diarrhoea relative to those who had—was based on a very small number of cases leading to low statistical power.

**Table 1:** Hospital admission for diarrhoea in the first 12 months of life and later mean blood pressure in adolescence and early adulthood (1982 Pelotas birth cohort)

	Systolic blood pressure (mm Hg)		Diastolic blood pressure (mm Hg)	
	Unadjusted	Adjusted*	Unadjusted	Adjusted*
Follow-up at age 15 years (4.9%)†				
Diarrhoea admission (n = 15)	119.0	119.6	70.5	71.8
No diarrhoea admission (n = 293)	114.3	114.0	68.1	67.2
p value for difference	0.14	0.11	0.39	0.14
Follow-up at age 18 years (6.4%)				
Diarrhoea admission (n = 37)	134.1	134.7	78.5	78.4
No diarrhoea admission (n = 544)	134.8	135.1	77.1	76.1
p value for difference	0.77	0.89	0.49	0.35
Follow-up at age 19 years (2.8%)				
Diarrhoea admission (n = 4)	104.8	91.8	64.8	52.0
No diarrhoea admission (n = 138)	113.6	113.9	71.7	71.7
p value for difference	0.09	0.01	0.15	0.14
Follow-up at age 23 years (6.5%)				
Diarrhoea admission (n = 73)	118.2	124.7	74.7	79.3
No diarrhoea admission (n = 1058)	117.3	123.0	73.6	77.4
p value for difference	0.63	0.35	0.41	0.22

*Adjusted for sex, age, height and body mass index (all at the time of blood pressure measurement), birthweight and gestational age, maternal education and family income.

†Prevalence of diarrhoea during first 12 months of life in parentheses.

In the largest analytical sample based on a re-survey of study members with data on diarrhoea status at 20 months and measured blood pressure at age 23 years (when, for the first time, attempts were made to contact the full cohort of men and women), the was again no evidence of a diarrhoea–blood pressure relationship. Thus, in 344 diarrhoea cases (8.7%) among 3944 study participants, there was no difference (diarrhoea cases vs diarrhoea-free; p value for difference) in systolic (122.1 vs 123.3 mmHg; p = 0.18) or diastolic (75.3 vs 75.9 mmHg; p = 0.33) blood pressure according to diarrhoea status in multiply adjusted analyses. All other diarrhoea–blood pressure associations were similarly null.

Finally, there was no suggestion that either type of water supply or sanitation facilities in early life, themselves related to diarrhoea occurrence in this cohort, was associated with later blood pressure at any point in follow-up (results not shown).

## DISCUSSION

In the present dataset we found little support for any link between diarrhoea in the first 12 or 20 months of life and later blood pressure. The very few results that attained statistical significance at conventional levels are likely to be chance findings given the large number of tests necessarily conducted in the course of these analyses. These findings accord with those from pooled analyses of cohorts from Hertfordshire (UK) where no relation was seen for nurse-recorded diarrhoea up to 5 years of age and blood pressure in old age.^5^ In that study, we also found no association of childhood diarrhoea with either non-fatal^5^ or fatal^6^ coronary heart disease in older age, a condition for which raised blood pressure is an important risk factor. The Pelotas study offers more statistical power than these analyses: the prevalence of diarrhoea in the first 12 months of life in the 1920s/1930s in Hertfordshire was low (3.4%)^5^ but generally higher herein (range 2.8–6.5%).

In the present study, hospital admissions for diarrhoea may be due to dehydration but also persistent diarrhoea and dysentery. It is not possible to know what proportion of study members were admitted for the former, which is of particular relevance to our programming hypothesis. Further investigation of its association, if any, with blood pressure is therefore required. As outlined elsewhere,^1^ ^5^ this includes, in humans, replication of the present study design using cases of hospitalisation for dehydration as utilised in one of the few positive studies,^1^ randomised trials of improved hygiene practices that reduce diarrhoea among infants (for instance, in low- and middle-income countries) and, in animals, trials of severe dehydration in early life on later blood pressure.

In conclusion, in the most comprehensive examination to date of the association between childhood diarrhoea and later blood pressure, we found no support for a relationship.

**What is already known on this subject**

Studies exploring the association between dehydration in early life and later blood pressure reveal equivocal findings and have low statistical power. We attempted to contribute to this evidence base, which is of particular importance in low- and middle-income countries, by examining the association in a cohort which offers both a higher prevalence of diarrhoea (a proxy for dehydration) and a greater number of serial blood pressure measurements than has previously been the case.

**What this study adds**

In the most comprehensive examination to date of the association between childhood diarrhoea and later blood pressure, we found no support for a relationship.
